# Synthesis and photochromism of catechol-containing symmetrical azobenzene compounds

**DOI:** 10.1098/rsos.211894

**Published:** 2022-06-08

**Authors:** Suju Fan, Yintung Lam, Liang He, John H. Xin

**Affiliations:** ^1^ Institute of Textiles & Clothing, The Hong Kong Polytechnic University, Hong Kong; ^2^ Shenzhen Research Institute, The Hong Kong Polytechnic University, Shenzhen, Hong Kong

**Keywords:** azobenzene, symmetrical structure, catechol, photochromism, adhesion

## Abstract

Symmetrical azobenzene derivatives with two catechol groups, 1d–4d, were synthesized as kinds of novel compounds, and the structures were confirmed using mass spectrometry and nuclear magnetic resonance spectroscopy. These compounds could attain photostationary state rapidly in solution upon UV irradiation, and their photochromism had good reversibility. Substituents and their positions on azobenzene chromophore had obvious influence on the maximum absorption and photochromic performances of these as-synthesized compounds. Electron-donating group on *ortho* positions could contribute to the redshift π–π* band. The sulfonamide group that is bonded to dopamine molecules and azobenzene rings caused a negligible n–π* transition of *cis* isomer, resulting in photobleaching upon UV irradiation. Among the four compounds, 4d had the strongest electron-donating *ortho*-methoxy substituents and lower planarity; thus its absorption could decrease more significantly upon UV irradiation of the same intensity, and its *cis*-to-*trans* conversion could be up to 63%. Furthermore, owing to the presence of catechol groups, 4d showed an effective affinity and adhesion to substrate, and on the surface of substrate, a weak colour change could be observed upon UV irradiation, but the reversibility was poorer than that in solution.

## 1. Introduction

Organic photochromic compounds have been widely studied in past decades because they have great applications in optoelectronic devices [1–7]. Under UV irradiation, they can reversibly transform from one isomer to another isomer via *cis*–*trans* isomerization, pericyclic reaction, intramolecular proton transfer or electron transfer [8–11]. Although many organic photochromic compounds have been developed, the most commonly studied compounds are classified into several types including azobenzenes, spiropyrans, spirooxazines, diarylethenes and naphthopyrans [8,12–17]. To fulfil different application requirements, structural modifications by the substitution of functional groups in these photochromic molecules were frequently used in many studies [3,4,15,18–22].

**Scheme 1.**
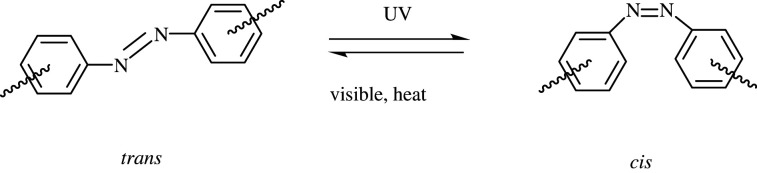
Reversible *trans*–*cis* isomerization of azobenzene derivatives.

Among various photochromic compounds, azobenzenes have gained extensive attention due to their easy synthesis, high sensitivity and rapid response. Azobenzenes can undergo a reversible *trans*–*cis* photoisomerization [23,24]. The thermodynamically stable *trans* isomer can be converted to the metastable *cis* isomer under UV exposure, while the recovery to the *trans* isomer can be promoted upon visible light or through thermal back reaction (scheme 1) [25]. In natural environment, *cis* forms can turn to *trans* forms spontaneously because of the higher energy of *cis* twist conformation [24]. Azobenzene has two absorption bands, of which one is located in the UV range with high intensity, corresponding to the π–π* electronic transition of *trans* isomer, and the other band has an extremely weak absorption in the visible region arising from symmetry-forbidden n–π* electronic transition in the *trans* isomers [23]. During the photoisomerization of *trans* into *cis*, the absorption in visible region enhances gradually until the isomerization achieves a photostationary state. This is because the n–π* electronic transition is allowed in the *cis* isomers, while the absorption in the near UV region becomes weaker. Apart from the change in absorption of UV–visible light, it is often accompanied by the changes of the planar conformation. The photochemical and photophysical behaviours are used for various applications such as photoswitching, information storage, nonlinear optics and biomaterials [26–34]. In recent years, to employ appropriate push–pull substituents to tune the photoisomerization of azobenzene toward visible light has attracted extensive attention [28,35,36]. For some azobenzene compounds with unsymmetrical electron-donating or electron-withdrawing groups, though their absorption shifted to longer wavelengths, side effects including short half-life of *cis* isomer, low *cis-to-trans* conversion and indistinct colour change were also caused because of the increased overlap of the π–π* band and n–π* band [20,35,37–39].

It has been reported that incorporating amide (–NHCO–) group on *para* positions of azo rings can reduce the height of the energy barrier for conversion, improving the *cis-to-trans* conversion [40]. In addition, some symmetrical azobenzene derivatives with same *ortho* substitutions in published reports [41–43] demonstrated good ability of photoswitching in solution and had a longer half-life of *cis* isomer. However, their affinity to substrates is poor and photochromic behaviours in solid applications is still unknown.

Herein, we report the synthesis of novel kinds of symmetrical azobenzene derivatives by bonding azobenzene and dopamine molecules covalently using sulfonamide (–NHSO–). They showed high conversion ratio, and their absorption arising from n–π* electronic transition was negligible in *cis* isomer, showing a certain degree of colour fading with UV irradiation. Their absorption and photochromic performances were fully studied. Based on our recent study, a catechol group was also incorporated into a commercial dye as an adhesive fixation anchor to enhance the application versatility on different kinds of substrates [44]. Continuing this interest, the application of the as-synthesized azobenzene compounds on solid material was also studied preliminarily.

## 2. Experimental

### 2.1. Material and instruments

Aniline-4-sulfonic acid, 6-aminotoluene-3-sulfonic acid, 2-chloroaniline-5-sulfonic acid, 2-methoxyaniline-5-sulfonic acid, sodium hypochlorite solution with 6–14% active chlorine, 98% oxalyl chloride, tetrabutylammonium fluoride (TBAF) solution (1.0 M in tetrahydrofuran (THF) containing *ca* 5% water) and other reagent-grade chemicals including 3,4-dihydroxyphenethylamine (dopamine), anhydrous sodium carbonate and anhydrous sodium sulfate were purchased from Shenzhen Dieckmann Technology Co. Ltd and used without further purification. Mass spectrometry with high and low resolution was conducted with a Waters mass spectrometer. Nuclear magnetic resonance (NMR) spectra were recorded on a Jeol ECZ500R (500 MHz) spectrophotometer. The absorption spectra were recorded on a UH5300 UV–visible spectrophotometer. UV irradiation tests were conducted on a TU1202 ultraviolet exposure table with a central wavelength of 312 nm. The colour depth and reflectance spectra of the film adhered with as-synthesized compound were conducted using a Datacolor 650 spectrophotometer. The UV–visible absorption spectrum of Nylon-6 (PA) film was recorded by a Varian Cary 300 UV–visible spectrophotometer.

### 2.2. Synthesis of bis-catechol azobenzene compounds

**Scheme 2.**
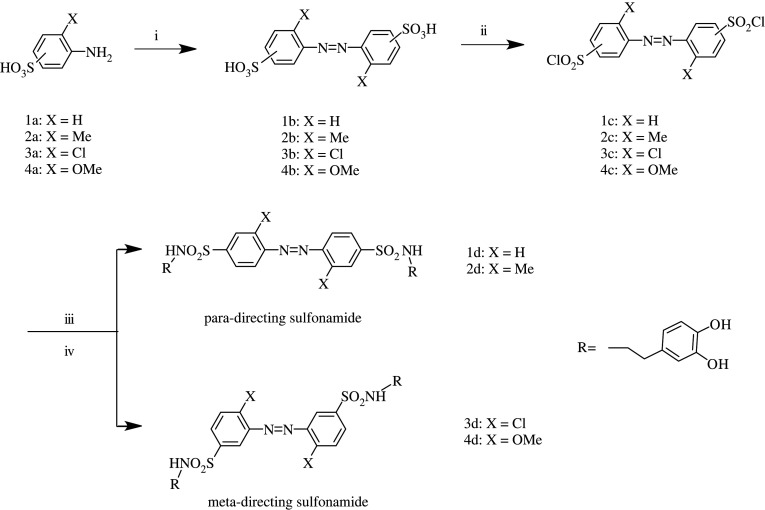
Synthesis route of the bis-catecholic azobenzene compounds. (i) NaClO/ice bath; (ii) (ClCO)_2_/reflux; (iii) TBDMS-dopamine/K_2_CO_3_; (iv) TBAF/THF.

The synthesis route of bis-catechol azobenzene compounds is shown in scheme 2, and the preparation of sample 4d is described in detail.

#### 2.2.1. Synthesis of 2,2′-bismethoxyazobenzene-5,5′-disulfonic acid (4b)

Compound 4a, 2-methoxyaniline-5-sulfonic acid (1.22 g, 6 mmol) and anhydrous sodium carbonate (1.5 g, 14 mmol) were added to 50 ml water under stirring. The resultant solution was cooled down in a cooling bath and 100 ml sodium hypochlorite solution (activated chlorine was about 6–14%) was added dropwise to it. After the addition, the solution was further stirred for 5 h in the bath. Then, the solution was neutralized with diluted hydrogen chloride to pH 7.0 and the precipitate was collected by filtration. After washing with deionized water and drying under vacuum at room temperature, the target was obtained as dark red solids (C_14_H_14_N_2_O_8_S_2_). Isolated yield was about 52.3%. HRMS (TOF, ESI^−^): *m/z*: [M − 2H]^2−^, calculated 200.0023, found 200.0020, error −1.5 ppm.

#### 2.2.2. Synthesis of 2,2′-bismethoxyazobenzene-5,5′-disulfonyl chloride (4c)

Compound 4b (1.01 g, 2.5 mmol) was added to 50 ml oxalyl chloride and refluxed overnight. After the reaction was over, the solution was poured into 500 ml ice water under stirring. Then, the mixture was filtered and washed with ice water until the filtrate became neutral. The obtained solid was dried under vacuum at room temperature. Isolated yield was about 43.6%. ^1^H NMR (chloroform-*d*): *δ* 8.30 (s, 2H, ArH), 8.17–8.20 (d, *J* = 11.49 Hz, 2H, ArH), 7.31–7.33 (d, *J* = 8.97 Hz, 2H, ArH), 4.20 (s, 6H, OCH_3_), as shown in electronic supplementary material, figure S5.

#### 2.2.3. Synthesis of catechol derivative: TBDMS-dopamine

A solution of dopamine hydrochloride (2.0 g, 10 mmol) in 50 ml dichloromethane was stirred in a water bath and *tert*-butyldimethylsilyl chloride (4.5 g, 30 mmol) in 20 ml dichloromethane was added. The water bath was replaced by an ice bath after 15 min. Triethylamine (5.0 g, 50 mmol) was added into the mixture and then stirred for 1 h. The resultant reaction solution was further stirred for about 20 h at room temperature. Then, 100 ml deionized water was added into the solution under stirring and the bottom organic layer was collected with a separation funnel. Then the collected solution was evaporated under vacuum to remove the solvent and the target was obtained as brown oil (C_20_H_39_NO_2_Si_2_) which was used in next reaction without any purification. HRMS (TOF, ESI^+^): *m/z*: [M + H]^+^, calculated 382.2593, found 382.2596, error 0.7 ppm.

#### 2.2.4. Synthesis of bis-catecholic compound (4d)

Compound 4c (0.22 g, 0.5 mmol), TBDMS-dopamine (0.57 g, 1.5 mmol), anhydrous sodium carbonate (0.16 g, 1.5 mmol) and anhydrous sodium sulfate (0.21 g, 1.5 mmol) were added into 25 ml ethyl acetate which was beforehand dried with anhydrous sodium sulfate. After stirring for 5 h at room temperature, the solvent was removed under vacuum and the residue was added into 25 ml tetrahydrofuran (THF). 2 ml TBAF solution was added, and the mixture was stirred for another 30 min at room temperature. Finally, the residue was obtained after removing THF and was purified by silica gel column chromatography using methanol/ethyl acetate/1,2-dichloroethane (1 : 1 : 5, v/v) as eluent to obtain 4d (C_30_H_32_N_4_O_10_S_2_). Isolated yield was about 45.2%. ^1^H NMR (DMSO-*d*_6_): *δ* 8.70–8.80 (dd, *J* = 23.36, 16.04 Hz, 4H, OH), 7.46–7.51 (m, 4H, ArH), 6.98–7.00 (d, *J* = 8.83 Hz, 2H, ArH), 6.95 (s, 2H, ArH), 6.63–6.65 (m, *J* = 7.96 Hz, 2H, NH), 6.52 (s, 2H, ArH), 6.36–6.38 (d, *J* = 7.95 Hz, 2H, ArH), 3.72 (s, 6H, OCH_3_), 2.55–2.59 (t, *J* = 6.76 Hz, 4H, CH_2_), 2.41–2.45 (t, *J* = 7.31 Hz, 4H, CH_2_), as shown in electronic supplementary material, figure S12. ^13^C NMR (DMSO-*d*_6_): *δ* 151.22, 145.46, 144.12, 143.52, 132.13, 129.88, 128.25, 119.82, 117.15, 116.54, 115.94, 112.82, 56.60, 44.77, 35.04, as shown in electronic supplementary material, figure S13. HRMS (TOF, ESI^−^): *m/z:* [M − H]^−^ calculated 671.1487, found 671.1491, error 0.6 ppm.

Compounds 1d, 2d and 3d were synthesized according to similar procedures, except 2-methoxyaniline-5-sulfonic acid (4a) was replaced by aniline-4-sulfonic acid (1a), 6-aminotoluene-3-sulfonic acid (2a), and 2-chloroaniline-5-sulfonic acid (3a), respectively.

1d (C_28_H_28_N_4_O_8_S_2_). Isolated yield was about 49.5%. ^1^H NMR (DMSO-*d*_6_): *δ* 8.79 (s, 2H, OH), 8.72 (s, 2H, OH), 8.08–8.10 (d, *J* = 8.51 Hz, 4H, ArH), 7.99–8.01 (d, *J* = 8.58 Hz, 4H, ArH), 7.88–7.91 (t, *J* = 5.68 Hz, 2H, ArH), 6.59–6.61 (d, *J* = 7.93 Hz, 2H, ArH), 6.52 (s, 2H, NH), 6.37–6.39 (d, *J* = 7.92 Hz, 2H, ArH), 2.90–2.96 (dd, *J* = 6.88, 13.74 Hz, 4H, CH_2_), 2.33–2.68 (m, 4H, CH_2_). ^13^C NMR (DMSO-*d*_6_): *δ* 153.96, 145.48, 144.14, 143.49, 129.73, 128.47, 123.95, 119.73, 116.44, 115.93, 45.00, 35.24. HRMS (TOF, ESI^−^): *m/z:* [M − H]^−^ calculated 611.1275, found 611.1278, error 0.5 ppm.

2d (C_30_H_32_N_4_O_8_S_2_). Isolated yield was about 38.1%. ^1^H NMR (DMSO-*d*_6_): *δ* 8.69 (s, 2H, OH), 8.63 (s, 2H, OH), 7.80 (s, 2H, ArH), *δ* 7.75–7.77 (d, *J* = 5.92 Hz, 2H, ArH), 7.69–7.71 (d, 2H, ArH), 7.65–7.66 (d, 2H, ArH), 6.55–6.56 (d, *J* = 7.79 Hz, 2H, ArH), 6.48 (s, 2H, ArH), 6.33–6.35 (dd, *J* = 1.43, 8.42 Hz, 2H, NH), 3.20–3.28 (t, *J* = 18.34 Hz, 4H, CH_2_), 2.86–2.91 (dd, *J* = 6.80, 14.28 Hz, 4H, CH_2_), 2.71 (s, 6, CH_3_). ^13^C NMR (DMSO-*d*_6_): *δ* 152.58, 145.56, 144.21, 143.12, 139.30, 130.05, 129.82, 125.65, 119.79, 117.22, 116.52, 115.98, 45.07, 35.34, 17.80. HRMS (TOF, ESI^−^): *m/z:* [M − H]^−^ calculated 639.1588, found 639.1587, error −0.1 ppm.

3d (C_28_H_26_Cl_2_N_4_O_8_S_2_). Isolated yield was about 57.3%. ^1^H NMR (DMSO-*d*_6_): *δ* 8.81 (m, 4H, OH), 7.98 (s, 2H, ArH), 7.55 (s, 2H, ArH), 6.64–6.66 (d, *J* = 7.88 Hz, 2H, ArH), 6.58–6.60 (d, *J* = 7.95 Hz, 2H, ArH), 6.51–6.53 (d, *J* = 7.54 Hz, 2H, ArH), 6.35–6.37 (d, *J* = 7.68 Hz, 2H, ArH), 5.30–5.32 (t, *J* = 4.81 Hz, 2H, NH), 2.68–2.72 (d, *J* = 19.32 Hz, 4H, CH_2_), 2.33 (s, 4H, CH_2_). ^13^C NMR (DMSO-*d*_6_): *δ* 145.46, 144.13, 132.70, 129.64, 119.71, 116.51, 116.32, 115.96, 44.95, 35.18. HRMS (TOF, ESI^−^): *m/z:* [M − H]^−^ calculated 679.0496, found 679.0497, error 0.14 ppm.

The mass fraction purity of compounds (1d–4d) could be indirectly determined using ^1^H NMR spectra obtained using methanol-*d*_4_ with TMS (tetramethylsilane) as NMR solvent. In particular, 20 mg 1d, 0.7 mg 2d, 20 mg 3d and 16 mg 4d were respectively dissolved in 0.4 ml methanol-*d*_4_ solvent with 0.03% (v/v) TMS under 30 min ultrasonication. The mass fraction purity could be then calculated using ^1^H NMR spectra of compounds (electronic supplementary material, figures S15–S18) and the following equation [45]:

2.1 $$w_{x}=\frac{I_{x}}{I_{s}}\times \frac{N_{s}}{N_{x}}\times \frac{M_{x}}{M_{s}}\times \;\frac{v}{m_{x}}\times v_{s},$$

where *w_x_* is the mass fraction of bis-catecholic azobenzene compounds, *v*_*s*_ is the known volume fraction content of the internal standard (TMS) in the NMR solvent of methanol-*d*_4_, *v* is the volume of methanol-*d*_4_ taken, *m_x_* are the masses of analyte; *I*_*x*_ and *I*_*s*_ are the integrals of the quantified signals of analyte and TMS, respectively; *N*_*x*_ and *N*_*s*_ are the number of ^1^H nuclei contributing to quantified signal of analyte and TMS, respectively; *M*_*x*_ and *M*_*s*_ are the molar masses of analyte and TMS, respectively (tables 1 and 2).

**Table 1.** The mass fraction purity of bis-catecholic compounds determined using ^1^H NMR spectroscopy.

compounds	1d	2d	3d	4d
purity	92.72%	89.70%	89.73%	91.64%

**Table 2.** The molecular structures of bis-catecholic azobenzene compounds.

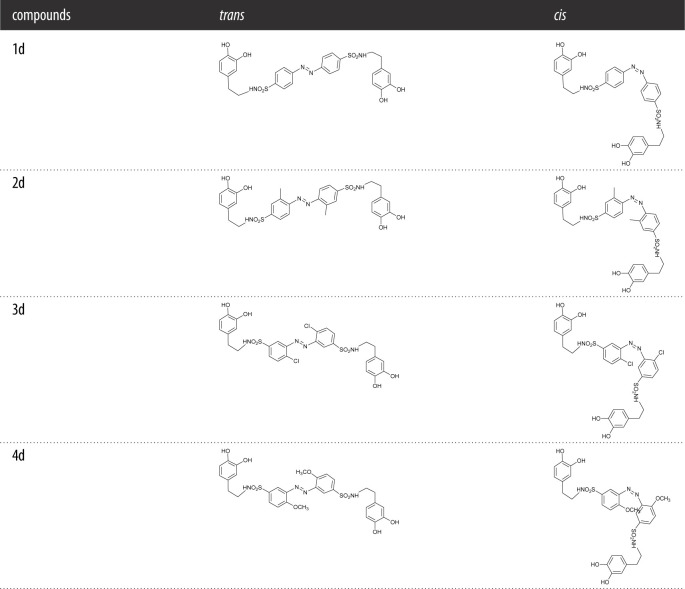

### 2.3. Spectral property

A stock solution of the catecholic azobenzenes was prepared in a mixture of ethanol with a concentration of 0.06 mol l^−1^ (for 1d and 2d) and 0.15 mol l^−1^ (for 3d and 4d). The UV intensity was about 1.0 mW cm^−2^, recorded using a Minolta luminance meter. The conversion ratio from *trans* isomer to *cis* isomer in the photostationary state of the catecholic azobenzenes was calculated according to equation (2.2) [46]:

2.2 $$\mathrm{Conversion}\;=\;\frac{\left(A_{0}-A_{t}\right)}{A_{0}}\times 100\text{\%},$$

where *A*_0_ and *A_t_* represent the absorbance at the maximum absorption wavelength of the catecholic azobenzenes before and after the UV irradiation, respectively.

### 2.4. Application on substate

The substrate used in this work was Nylon-6 (PA) film, and it was soaked with ethanol before use. Substrate was immersed into 0.5% (w/v) as-synthesized compound solution in ethanol at room temperature for 24 h. Then, the substrate was taken out from the solution, washed with water, and then air-dried. Compounds 4d, 4b and a modified azobenzene (4*β*) were used for studying the effect of catechol groups. The synthesis and characterization of 4*β* are provided in the electronic supplementary material.

## 3. Results and discussion

### 3.1. Synthesis of symmetric and catecholic azobenzenes

**Scheme 3.**
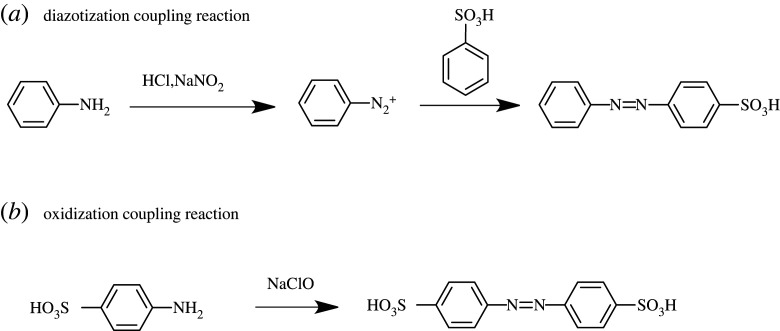
The comparison of (*a*) diazotization coupling reaction and (*b*) oxidization coupling reaction.

Azobenzene compounds are often synthesized through a diazotization coupling reaction [47–50]. These as-synthesized azobenzene compounds usually have asymmetric structures (scheme 3*a*). Symmetric azobenzene compounds are difficult to obtain using this reaction. In this study, we therefore adopted oxidization coupling reaction for synthesizing symmetric azobenzene compounds [51–53], where aromatic amines can self-couple to form azo bonds (scheme 3*b*). Compared to the oxidation agents and chlorination reagents used in previous reports [54–60], sodium hypochlorite with low cost, easy availability and proper oxidization ability [53] and oxalyl chloride which has mild reactivity and is easy to be after-treated [61] were used for both oxidation and chlorination. *tert*-Butyldimethylsilyl chloride was used to protect the catecholic group in dopamine molecule because it has a moderate stability and can easily be removed in the presence of TBAF under mild conditions after reaction [62–65].

### 3.2. Characterization of catecholic azobenzenes

The structures of the catecholic azobenzenes (1d, 2d, 3d and 4d) were established with the aid of mass spectral data and ^1^H NMR and ^13^C NMR analyses. The relevant spectra are provided in the electronic supplementary material.

**Figure 1.**
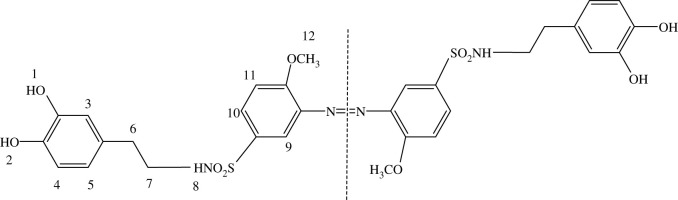
The proton distribution on the symmetrical molecular structure of 4d.

Typically, the ^1^H NMR spectrum of 4d was representatively analyzed in detail. The molecular structure of 4d and its proton distribution are shown in figure 1. Because of the special symmetrical structure, the spectrum shown in electronic supplementary material, figure S12, contains a coupling peak at *δ* 8.70–8.80 ppm for the two hydroxyl groups of the catechol units (H1 and H2). The multiplets at *δ* 7.46–7.51 ppm were attributed to the aromatic protons of azobenzene (H11 and H10), while doublets at *δ* 6.98–7.00 ppm were attributed to aromatic protons of the catechol units (H4), and the singlet at *δ* 6.95 ppm arises from the other aromatic protons of azobenzene (H9). The singlet at *δ* 6.52 ppm and the doublets 6.36–6.38 ppm arose from the aromatic protons of the catechol units (H3 and H5), respectively. The multi-peak at *δ* 6.63–6.65 ppm was attributed to imine protons (H8) which had a coupling interaction with the adjacent CH_2_ groups (H7). The singlet at *δ* 3.72 ppm was attributed to the protons of methoxy groups (H12). The triplets at *ca* *δ* 2.55–2.59 ppm and the peaks at about *δ* 2.41–2.45 ppm were attributable to aliphatic protons of H6 and H7, respectively. Other obvious peaks, multi-peak at *ca* *δ* 4.05 ppm probably arose from the solvent of ethyl acetate used in the process of synthesis and purification. It is worth noting that the peaks for H6 and H7 at *ca δ* 2.50 ppm were obviously interfered by the solvent residual peak. In order to verify the peaks, methanol-*d*_4_ was used as solvent for ^1^H NMR measurement again. As shown in electronic supplementary material, figure S18, its spectrum clearly showed the two triplets with the correct ratio of integral area at *δ* 3.04–3.07 and *δ* 2.56–2.59 ppm respectively, which confirmed the existence of H6 and H7. According to these results, 4d was successfully synthesized.

Similarly, the chemical structures of 1d, 2d and 3d were fully confirmed with these analyses.

### 3.3. Spectra

**Figure 2.**
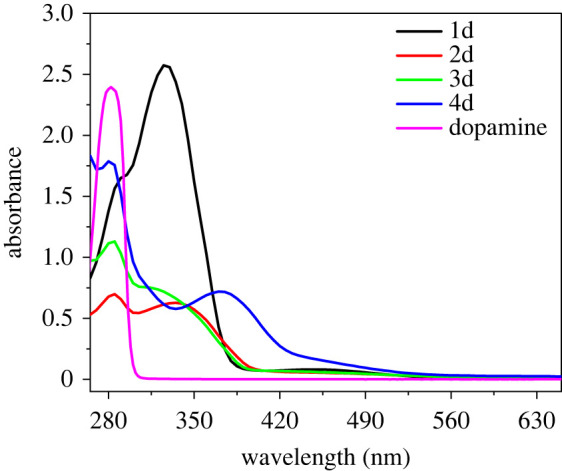
UV–visible spectra of the bis-catecholic azobenzene compounds (concentration: 0.15 mol l^−1^) and dopamine (concentration: 0.5 mmol l^−1^) in ethanol.

The absorption spectra of the as-synthesized azo compounds in ethanol are shown in figure 2. It could be seen that there is strong adsorption at *ca* 282 nm due to the presence of catechol moiety of the four azobenzene compounds [66]. The maximum absorptions (*λ*_max_) of 1d, 2d and 3d were located respectively at *ca* 325 nm, *ca* 335 nm and *ca* 315 nm, while 4d had a much larger *λ*_max_, and bathochromic shift to *ca* 370 nm. Compared with 1d, 2d was substituted with methyl groups and had a higher electron cloud density, resulting in slight bathochromic shifts of its *λ*_max_. Weak electron-withdrawing chlorine atom on 3d contributed to its hypsochromic shift. While the methoxy group on 4d donated more electrons to chromophores than methyl group [67,68], resulting in its more obvious bathochromic shifts. The molar absorption coefficient of as-synthesized compounds is shown in table 3. The presence of rotational substituents on *ortho* positions of azo rings worsens the planarity of 2d, 3d and 4d, and thus their molar absorption coefficients are lower than that of 1d [69].

**Table 3.** The molar absorption coefficients (*ε*) of the bis-catechol azobenzene compounds in ethanol.

compounds	1d	2d	3d	4d
*ε* (l mol^−1^ cm^−1^)	1.58 × 10^4^	4.02 × 10^3^	5.10 × 10^3^	4.83 × 10^3^

Their *λ*_max_ in other solvents are also listed in table 4, and the *λ*_max_ of these compounds were similar in polar and less polar solvents, which indicated that their absorption showed no obvious dependence on solvent polarity. The absorption spectra of these compounds in methanol, *N*,*N*-dimethylformamide (DMF) and acetone can be found in electronic supplementary material, figure S20.

**Table 4.** The maximum absorption wavelengths of bis-catecholic azobenzene compounds in different solvents.

compounds	ethanol (nm)	methanol (nm)	DMF (nm)	acetone (nm)
1d	325	328	330	332
2d	335	333	338	342
3d	315	314	318	338
4d	370	373	376	374

### 3.4. Photochromic performance

**Figure 3.**
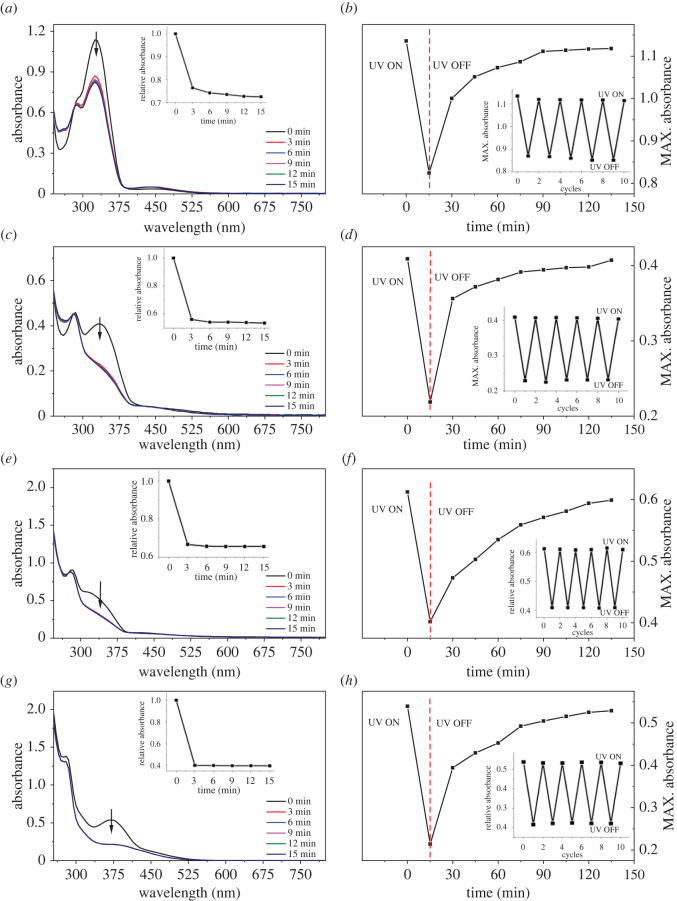
The responses to UV radiation (*a*,*c*,*e*,*g*) and photochromic recovery (*b*,*d*,*f*,*h*) of the bis-catecholic azobenzene compounds: (*a*,*b*), 1d; (*c*,*d*) 2d; (*e*,*f*) 3d; (*g*,*h*) 4d.

**Figure 4.**
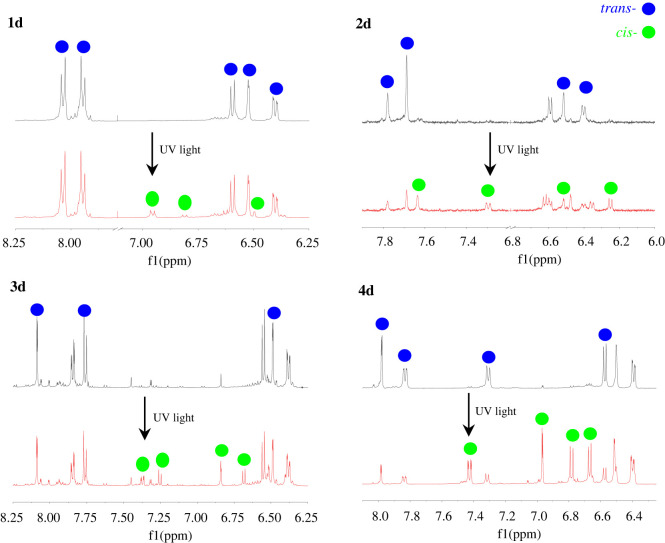
Partial ^1^H NMR spectra of bis-catecholic azobenzene compounds in methanol-*d*_4_ before and after UV irradiation.

The responses to UV radiation of these azobenzene compounds were firstly investigated, as shown in figure 3*a*,*c*,*e*,*g*. Under initial state, the azobenzene compounds existed mainly in the form of *trans* isomers, and the absorption bands of 1d, 2d, 3d and 4d arising from π–π* electronic transition could be found at 325 nm, 335 nm, 315 nm and 370 nm, respectively. These absorption bands rapidly decreased upon UV irradiation for 3 min, and any further increase in irradiation time had no obvious effect on the absorption. This indicated that the interconversion of two isomers in solution reached equilibrium, achieving photostationary state. Some research has pointed out that n–π* electronic transition is allowed in *cis* isomers [70,71]. However, among the four compounds, only *cis* isomers of 1d had a slight increase in visible region at *ca* 425 nm after UV irradiation. The possible reasons were that two sulfonamide groups bonding to dopamine molecule at the other end had strong electron-withdrawing ability and this decreased the electron density at the nitrogen atoms of azobenzene chromophore, leading to a lower chance of electron transition; and that in *cis* isomers, *ortho* substituents, especially the group containing a lone pair of electrons, greatly increased electronic repulsion centred on the N = N unit, leading to a worse planarity of the molecules. Thereby, the absorption in visible region was negligible. The *trans*-to-*cis* conversion ratios of 1d, 2d, 3d and 4d were *ca* 26.6%, 46.5%, 27.6% and 63.6%, respectively. This might be caused by the electron-donating ability of substituted groups. Compared to 1d, the electron density of 2d increased through the hyperconjugation of methyl group and its conversion ratio had a sharp increase. For 3d, the negative inductive effect of chlorine atom cancelled the effect of conjugation, and thus its conversion remained almost unchanged. While the methoxy group on 4d was the strongest electron-donating substituent among the four (H, CH_3_, Cl, OCH_3_), so its conversion ratio was highest in these compounds, and was better than a previously reported result [40]. Partial ^1^H NMR spectra of compounds (1d–4d) before and after UV irradiation are shown in figure 4, in which the aromatic signals showed varying degrees of changes. Taking 4d as an example, there were six types of aromatic signals with chemical shifts of *δ* 6.4–8.0 ppm before UV irradiation, and these were assigned to the aromatic protons of *trans* isomers. After UV irradiation, new sets of aromatic signals appeared at *δ* 7.41–7.44, 6.96–6.97, 6.77–6.99, and 6.66–6.68 ppm due to the formation of the *cis* isomer. Similar results could also be found from the spectra of 1d, 2d and 3d. Among the four compounds, the signal changes of 4d were the most obvious, followed by 2d, and those of 1d and 3d were less obvious. This result was consistent with their *trans*-to-*cis* conversion ratio.

Reversibility and stability are critically important parameters for the application of photochromic materials. The photochromic reversibility and stability of these azobenzene compounds are shown in figure 3*b*,*d*,*f*,*h*. The solutions of these azobenzene compounds were irradiated by UV for 15 min, and their absorbances thus decreased to the lowest values, indicating the structural transformation from *trans* to *cis* form. After the removal of UV irradiation, the absorbances gradually increased and recovered back to the initial values in *ca* 135 min, which meant the *cis* form was converted back to the *trans* form spontaneously in natural environment. These results showed that the photochromic performance of these azobenzene compounds had good reversibility. Moreover, the photoisomerization process of *trans*-to-*cis*-to-*trans* could be repeated without significant loss of reversibility for five cycles, as shown in the insets in figure 3*b*,*d*,*f*,*h*.

### 3.5. Application on substrate

**Figure 5.**
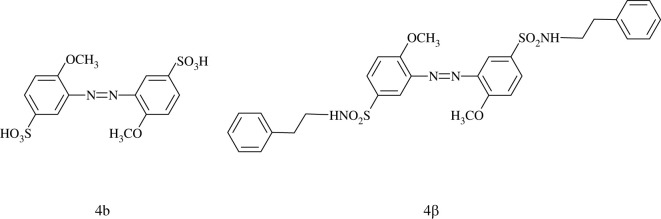
The molecular structures of 4b and 4*β*.

**Figure 6.**
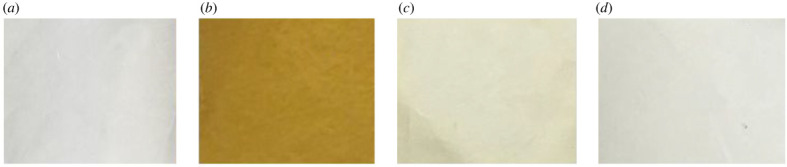
The photographic images of PA film deposited with different compounds. (*a*) Blank film, (*b*) 4d, (*c*) 4*β*, (*d*) 4b.

Under the influence of different factors like substitutions and molecular planarity, 4d has the highest conversion ratio and the most obvious photochromic changes among these as-synthesized compounds. Thus, 4d was selected as a representative example to investigate the functionality of catechol groups in azobenzene derivatives. Compound 4*β* was developed for this comparative study, it being 4b modified with *β*-phenylethylamine which possessed a similar structure to dopamine but without catechol groups (figure 5). The deposition of 4d on Nylon-6 (PA) film was then compared with that of 4b and 4*β* (figure 6). The appearance of film treated with 4d showed obvious colour while the other two had a colour similar to the blank film. This might imply that the catechol groups on 4d could act as anchors for substrate adhesion, just as marine mussels adhered to rocks [64,72]. By contrast, compounds 4b and 4*β* had no such functional groups, so they could not adhere to PA film.

**Figure 7.**
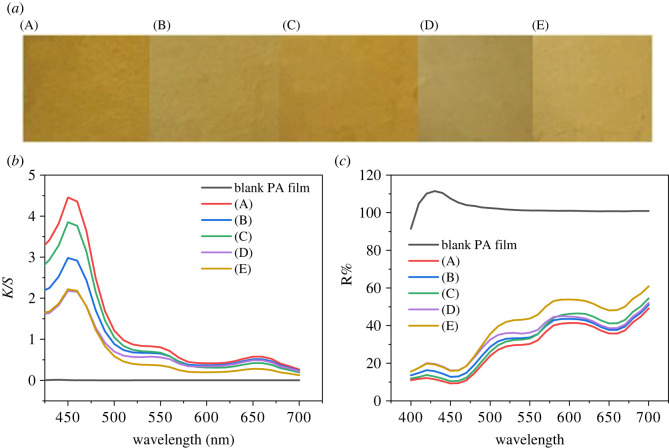
(*a*) Photographic images, (*b*) *K/S* curves and (*c*) reflectance spectra of PA film treated with 4d before and after UV irradiation: (A) before UV irradiation, (B) under UV irradiation for 2 h, (C) 1 day after removal of UV source, (D) under UV irradiation for 2 h again, (E) 1 day after removal of UV source again.

The PA film deposited with 4d was exposed to UV light for 2 h and then allowed to undergo spontaneous back reaction for 1 day. The photographs, *K/S* values and reflectance spectra of the PA film are shown in figure 7, and the UV–visible absorption spectra of (A) to (E) can be found in electronic supplementary material, figure S21. In the first cycle, the PA film showed a certain degree of photobleaching upon UV irradiation, and it could return to nearly the original colour after removing the UV source for 1 day. In the second cycle, the photobleaching of the film was more obvious after UV irradiation, but it could not recover after removing the UV source. Especially, the *K/S* curve of (E) had little difference from that of (D), and its reflection curve did not go down as that of (C) did. The weak photochromic change and poor reversibility of 4d shown on PA film were probably caused by the geometric isomerization of azobenzene [10], and on solid substrate it could not move as freely as it did in solution owing to space limitation. However, this study has successfully demonstrated the potential of catechol-containing photochromic compounds for both photochromism and versatile adhesion. This is beneficial for the future development of photochromic compounds in various fields.

## 4. Conclusion

Symmetrical azobenzene compounds were synthesized by bonding azobenzene and dopamine molecules covalently using sulfonamide (–NHSO–). They had maximum absorption near 325 nm, and these compounds could attain their photostationary state rapidly under UV irradiation in solution. In addition, they had a relatively high *cis-to-trans* conversion, and good photochromic behaviours in terms of reversibility and stability. Though the introduction of dopamine molecules had no obvious impact on bathochromic shifting, the sulfonamide bonding to dopamine molecule and the substituents on *ortho* position of the azobenzene rings might weaken the absorption arising from n–π* transitions in *cis* isomers. This resulted in observable colour fading under UV irradiation, especially for 4d with the strongest electron-donating substituent and lower molecular planarity. Furthermore, 4d showed a good affinity and adhesion to substrate due to the presence of catechol groups. Though the reversibility on the surface of treated substrate was poorer than that in solution, a slight colour change could be observed upon UV irradiation. Thus, these novel bis-catecholic azobenzene derivatives have a potential application in both photochromism and versatile adhesion, which also promotes the development of catechol-containing photochromic compounds in various fields.

## Data Availability

The data and spectra supporting this article are available in the electronic supplementary material, supporting information. The stick models of bis-catholic compounds are shown in electronic supplementary material, S1 [73]. The characterization spectra are shown in electronic supplementary material, S2–S19. The UV–visible spectra of bis-catecholic compounds in different solvents are shown in electronic supplementary material, S20. the UV–visible absorption spectra of Nylon-6 (PA) film treated with 4d are shown in electronic supplementary material, S21.
